# Quantitative analysis of H_2_O_2_ transport through purified membrane proteins

**DOI:** 10.1016/j.mex.2020.100816

**Published:** 2020-02-20

**Authors:** Hao Wang, Stefan Schoebel, Florian Schmitz, Hansong Dong, Kristina Hedfalk

**Affiliations:** a Department of Plant Pathology, Nanjing Agricultural University, 1 Weigang, Nanjing 210095, China; b Department and Chemistry and Molecular Biology, Gothenburg University, Box 462, 405 30 Göteborg, Sweden

**Keywords:** Proteoliposomes, Hydrogen peroxide, Aquaporin, Permeability, Membrane protein structure

## Abstract

Hydrogen peroxide (H_2_O_2_) is an important signal molecule produced in animal and plant cells. The balance of H_2_O_2_ between the intra- and extracellular space is regulated by integral membrane proteins, which thereby modulate signaling. Several methods have been established to analyze aquaporin mediated transport of H_2_O_2_ in whole cells with the intrinsic limitation that the amount of protein responsible for a certain activity cannot be standardized. As a consequence, the quantification of the transport and specific activity is difficult to extract making it problematic to compare isoforms and mutated variants of one specific target. Moreover, in cell-based assays, the expression of the target protein may alter the physiological processes of the host cell providing a complication and the risk of misleading results. To improve the measurements of protein based H_2_O_2_ transport, we have developed an assay allowing quantitative measurements.

• Using purified aquaporin reconstituted in proteoliposomes, transport of H_2_O_2_ can be accurately measured.
• Inside the liposomes, H_2_O_2_ catalyzes the reaction between Amplex Red and horseradish peroxidase (HRP) giving rise to the fluorescent product resorufin.
• Analysing pure protein provides direct biochemical evidence of a specific transport excluding putative cellular background.

## Graphical abstract

**Figure:**
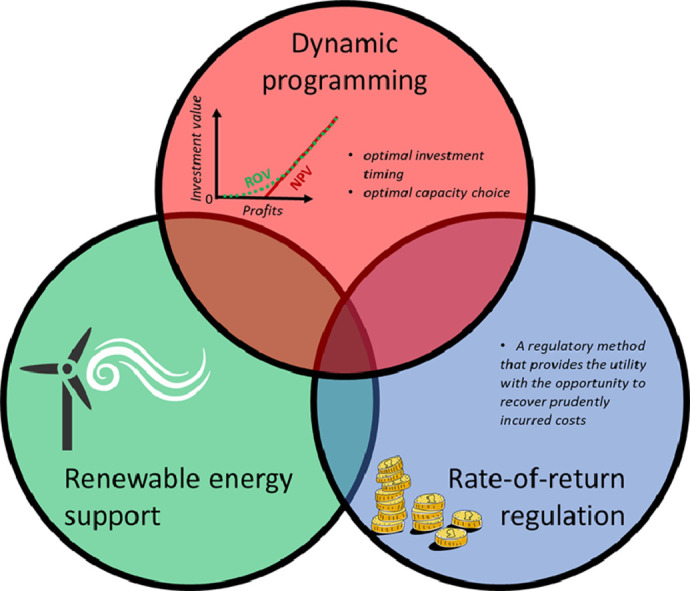

**Table:** Specification table

Subject Area:	Biochemistry, Genetics and Molecular Biology
More specific subject area:	Function and specificity of membrane proteins
Method name:	Quantitative assay for H_2_O_2_ permeability of aquaporins
Name and reference of original method:	Piwonski, H. M., Goomanovsky, M., Bensimon, D., Horovitz, A. & Haran, G. Allosteric inhibition of individual enzyme molecules trapped in lipid vesicles. *Proc Natl Acad Sci U S A***109**, E1437-1443, doi:10.1073/pnas.1116670109 (2012).
Resource availability:	Plate reader

## Overview of the method

**Fig. 1:**
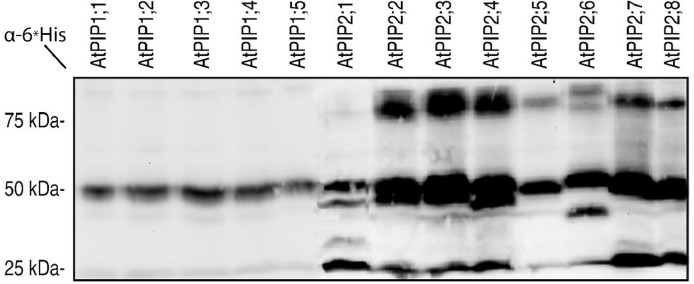
Recombinant production of aquaporins in yeast for functional studies. Immunoblot showing the variation in production levels of the 13 AtPIPs expressed in yeast cells using the vector of pYES2. All AtPIP isoforms are fused to a C-teminal 6xHis tag and detected using an anti-His antibody. *Figure adapted from Wang et al 2020.*

Cell-based methods for aquaporin mediated hydrogen peroxide transport include qualitative growth assays using yeast cells combined with fluorescent dyeing and fluorescent probe. These measurements rely on the expression of the channel proteins in the host cells, and, most importantly, proper localization of the recombinant protein to the cellular membrane [1], [2], [3], [4], factors that are difficult to fully control and standardize. Noteworthy, recombinant production of the 13 highly homologues plasma membrane intrinsic proteins (PIPs) isoforms from *Arabidopsis thaliana* in *Saccharomyces cerevisiae* using the expression vector pYES2 [5] resulted in major variations in protein levels (Fig. 1). In conclusion, even when using the same promoter and growth conditions, comparing transport efficiencies and specificities for the AtPIP isoforms was non-trivial using the cell-based system [6]. Furthermore, working with cell-based systems, there is a substantial risk that background reactions within the complex intracellular environment of the host cell interfere with reaction of interest and influence the final results, an aspect that is also pronounced in less complex cells like yeast. In addition, the recombinant expression as such, aiming at producing a foreign protein within the cellular environment, might have a negative impact on endogenous physiological processes which could also interfere with the analysis giving misleading results.

**Fig. 2:**
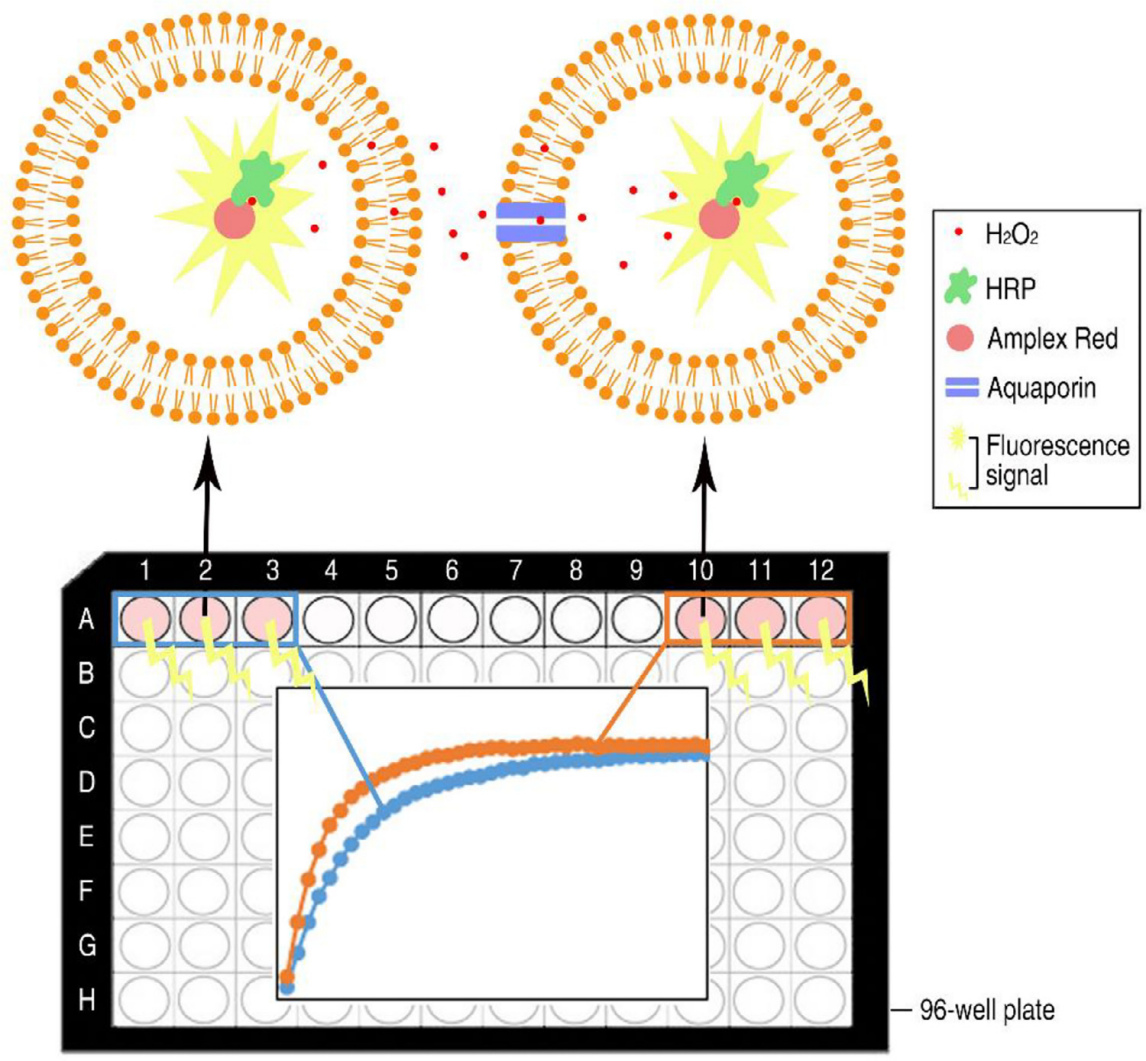
Schematic figure of the proteoliposome assay with reconstituted aquaporin for functional analysis of H_2_O_2_ transport using a 96-well format. In order to provide the detection system for H_2_O_2_ transported via the lipid bilayer, Horseradish peroxidase (HRP) and Amplex Red are encapsulated in the lipid vesicles. The aquaporin mediated transport (orange trace) is more efficient than simple diffusion (blue trace) giving rise to a faster increase in the fluorescent signal as compared to background. *Figure adapted from Wang et al 2020.*

To avoid possible complications and lack of reproducibility connected to the use of cell-based systems, a biochemical method for direct evidence of H_2_O_2_ transport mediated by selected membrane proteins has been developed using artificial liposomes as host system. It has previously been shown that the enzymatic reaction of horseradish peroxidase (HRP) can be assayed encapsulated in a lipid vesicle [7] and the method presented here is a development of this assay where integral membrane proteins reconstituted into the liposome have been included. In brief, the method relies on liposomes having HRP and Amplex Red (Invitrogen) trapped inside and, at time zero, a defined amount of H_2_O_2_ is added on the outside. By balancing the concentrations of internal HRP and Amplex Red, respectively, as well as optimizing the external concentration of H_2_O_2_, the transport efficiency of proteoliposomes as compared to liposomes can be evaluated. The externally added H_2_O_2_ penetrates the lipid membrane of the liposomes by simple diffusion, but importantly, the transport is more efficient via the aquaporin channel. When H_2_O_2_ reaches the inside of the vesicle, HRP catalyzes the reaction of Amplex Red and H_2_O_2_ forming the highly fluorescent product resorufin which could be quantified using a plate reader (Fig. 2). The main principle of the method is that a more efficient H_2_O_2_ transport over the membrane gives rise to a higher fluorescence. Thereby, the transport of different aquaporin isoforms can be quantified and accurately compared using empty liposomes as a reference. In principle, there is nothing hindering the evaluation of other membrane proteins using this method, providing a general method for H_2_O_2_ transport using proteoliposomes.

## Experimental procedure

### Protein purification and standardization of the amount

Purified protein is required for the reconstitution experiment. Noteworthy, purification of integral membrane proteins in high yields is challenging in itself, and it is well established that eukaryotic membrane proteins are the high-hanging fruits for recombinant protein production. Yeast, and especially the methylotrophic strain *Pichia pastoris*, has been shown to be a reliable host for challenging membrane protein targets [8]. High level production of stable protein is a prerequisite for subsequent purification, where the choice of detergent and purification protocol has to be optimized to suit each specific target. For AtPIP2;4, specifically, the key steps for the optimized protein purification procedure [6] is described below.

(a) For large scale production, *culture Pichia pastoris* transformed with recombinant aquaporin fused with a C-terminal 8xHis-tag in fermentor, harvest cells and keep at -20°C for future purification.
(b) For purification, thaw cells and resuspend them in buffer (20 mM Tris, 20 mM NaCl). For *cell breakage*, grind the cell resuspension in a bead beater (e.g Bio Spec) for 12 × 30 s, with 30 s cooling between each run. Collect unbroken cells by centrifugation.
(c) Collect the *membrane fraction* by ultracentrifugation (200,000 × g, 30 min, 4°C) and resuspend the membrane pellet in buffer (20 mM Tris-HCl, 200 mM NaCl, 10% glycerol).
(d) Perform a *detergent screen* to find the best solubilizing agent for the target of interest with respect to efficiency without compromising with protein function. It is recommended to screen the following detergents: 3-[(3-cholamidopropyl) dimethylammonio]-1-propanesulfonate (CHAPS), n-Dodecyl-β-DMaltopyranoside (DDM), n-Decyl-β-D-Maltopyranoside (DM), FosCholine-12 (fc12), n-Dodecyl-N,N-Dimethylamine-N-Oxide (LDAO), Lauryl Maltose Neopentyl Glycol (MNG), Decyl Glucose Neopentyl Glycol (NG) and n-Octyl-β-D-Glucopyranoside (β-OG). For the detergent screen, each detergent is added drop-wise to the membrane resuspension and the final mixture is incubated at 4°C for 2 h with rotation. Non-solubilized membranes are spun down at 160,000g for 30 min at 4°C and supernatant as well as pellet are collected for immunoblot analysis to evaluate the efficiency of each detergent.
(e) For *purification*, add 10 mM Imidazole to the solubilized material and incubate the solution with Ni-NTA agarose for 3 h at 4°C. Elute the target aquaporin in elution buffer (20 mM HCl, 200 mM NaCl, 10% glycerol, detergent, 300 mM imidazole) and concentrate the final protein sample using e.g VivaSpin 100 kDa concentration tube (Sartorius Stedim Biotech GmbH).
(f) If increased purity is required, a gel filtration step is advised using e.g a Superdex200 increase 10/300 GL column (GE Healtcare).

For proper comparison of the transport efficiencies, it is critical to standardize the amount of protein in each reconstitution experiment. In the case of H_2_O_2_ transport measurements via aquaporins, the concentration for each protein preparation was determined using the BCA protein assay or absorbance measurement (280 nm) using the protein specific extinction coefficients, and the concentration was then adjusted to 0.3 mM for each target.

### Preparation of aquaporin-liposomes with encapsulated HRP

All reagents and equipment needed for the reconstitution experiment are listed in Box 1. It is highly recommended **to prepare all the reagents in advance.**

Box 1 **Reagents and equipment⁎**

**Table:** 

**Lipids** (*E. coli* Polar Lipid Extract, Avanti Polar Lipids. inc)
**Reconstitution buffer** (50 mM NaCl, 50 mM Tris pH 8.0, with or without 1 mM EDTA)
**10% OG** (n-Octyl-β-D-Glucoside, Anatrace)
**1 M NaCl**
**0.5 M Tris** pH 8.0
**200 U/ml HRP** (type II, Sigma Aldrich)
**GF buffer** (the buffer used to elute the protein in the last purification step, in the case of aquaporins, 200 mM NaCl and 20 mM Tris pH 8.0 was used).
**Bio-Beads** (Bio-Beads® SM-2 Hydrophobic absorbents, Bio-Rad)
**S400HR Column** (GE Healthcare)
**Ultracentrifuge** (Beckman)
**Roller table** (kept at 4°C)

⁎ Here we specify material successfully used for the aquaporin containing liposomes in Wang et al 2020, which does not exclude that alternatives could work equally well.

Alt-text: Unlabelled box

The experimental procedure for reconstitution of aquaporin in proteoliposomes is listed below as a-i. It is strongly advised to be thorough in the pipetting procedure so that equal amounts are added in each sample. Further, to avoid disruption of the preparation it is important to treat the samples as gentle as possible once the vesicles are formed.

(a) Mix lipid powder with Reconstitution buffer to a concentration of 25 mg/mL. Aliquot the homogenized mixture into 2 mL tubes, 160 µL in each. The aliquots could be stored at 4°C for up to two weeks.
(b) For the reconstitution experiment, add compounds to the 2 mL tubes as follows:

**Table:** 

	Liposomes	Proteoliposomess
Lipid (25 mg/mL)	160 µL	160 µL
ddH_2_O	610 µL	610 µL
1 M NaCl	42 µL	42 µL
0.5 M Tris pH 8.0	84 µL	84 µL
Mix gently		
10% OG	100 µL	100 µL
Mix them and let stand for 5 min, now the mixture should be clear.		
HRP (200 U/mL)	2 µL	2 µL
GF buffer	6.5 µL	-
Aquaporin (0.3 mM)	-	6.5 µL
Mix them gently and wait for 5 min		
Bio-Beads*	300 mg	300 mg

*Note: bio-beads should be activated by methanol, washed with mQ water two times and finally equilibrated with reconstitution buffer.
(c) Incubate the mixtures from step b) at 4°C for 3 h with rotation.
(d) To get rid of the bio-beads, centrifuge the mixture (13000xg, 4°C for 5 min) using a 0.22 µm spin filter.
(e) Mix the flow-through with 5 mL Reconstitution buffer (with 1 mM EDTA, all the reconstitution buffer used from this point should have EDTA added). Collect liposomes by ultracentrifugation (140,000xg, 4°C for 45min).
(f) Remove the supernatant and wash the liposomes with 5 mL Reconstitution buffer. Collect the liposomes by ultracentrifugation (140,000xg, 4°C for 45min). Discard the supernatant.
(g) Resuspend the liposomes in 200 µL Reconstitution buffer by gentle mixing. *Please note that the liposomes are fragile and break easily*.
(h) Wash residual HRP outside the liposomes away using the S400HR Column (GE Healthcare) [7], 100 µL of the liposome solution is spun at 700xg, 4°C for 30s.
(i) The final flow through is gently mixed with Reconstitution buffer, 500 uL. The liposomes/proteoliposomes are now ready for use.

### Hydrogen peroxide transport assay

The actual measurements of hydrogen peroxide is performed in 96-well plates (Fig. 2) and the detailed procedure is listed in a-e below:

(a) Add 0.6 µL of Amplex Red (AR, Thermo Fisher SCIENTIFIC) stock solution (100 mM) to each of the prepared liposome samples and mix gently. In order to prepare a negative control, blocking the aquaporin transport with mercury, additionally 0.9 µL 200 mM HgCl_2_ is added by gentle mixing.
(b) Add the liposome/proteoliposome mixture to black Matriplate 96 glass bottom plate (Brooks LIFE SCIENCE SYSTEMS), 150 µL in each well. For each sample, three repeats are prepared in individual wells. The plate is incubated at room temperature for 20 min before measurements.
(c) Meanwhile, prepare the H_2_O_2_ solution, 200 µM in Reconstitution buffer (0.2 µL 30% H_2_O_2_ to 10 mL Reconstitution buffer).
(d) Add the 200 µM H_2_O_2_ solution to the liposome/proteoliposome mixture, 50 µL in each well, using a multipipette.
(e) For fluorescent measurements, the plate is immediately put into a plate reader (e.g POLARstar Omega, BMG TECH). The fluorescent signal from the oxidation of Amplex Red by HRP and H_2_O_2_ inside the liposomes is collected for 40 cycles with an interval of 2 min, using the wavelength of 544 nm and 590 nm for excitation and emission, respectively. Since H_2_O_2_ penetrates the liposomes very fast, it is recommended to set up the program before adding H_2_O_2_.

## Method validation

### Optimizing the concentration of H_2_O_2_

**Fig. 3:**
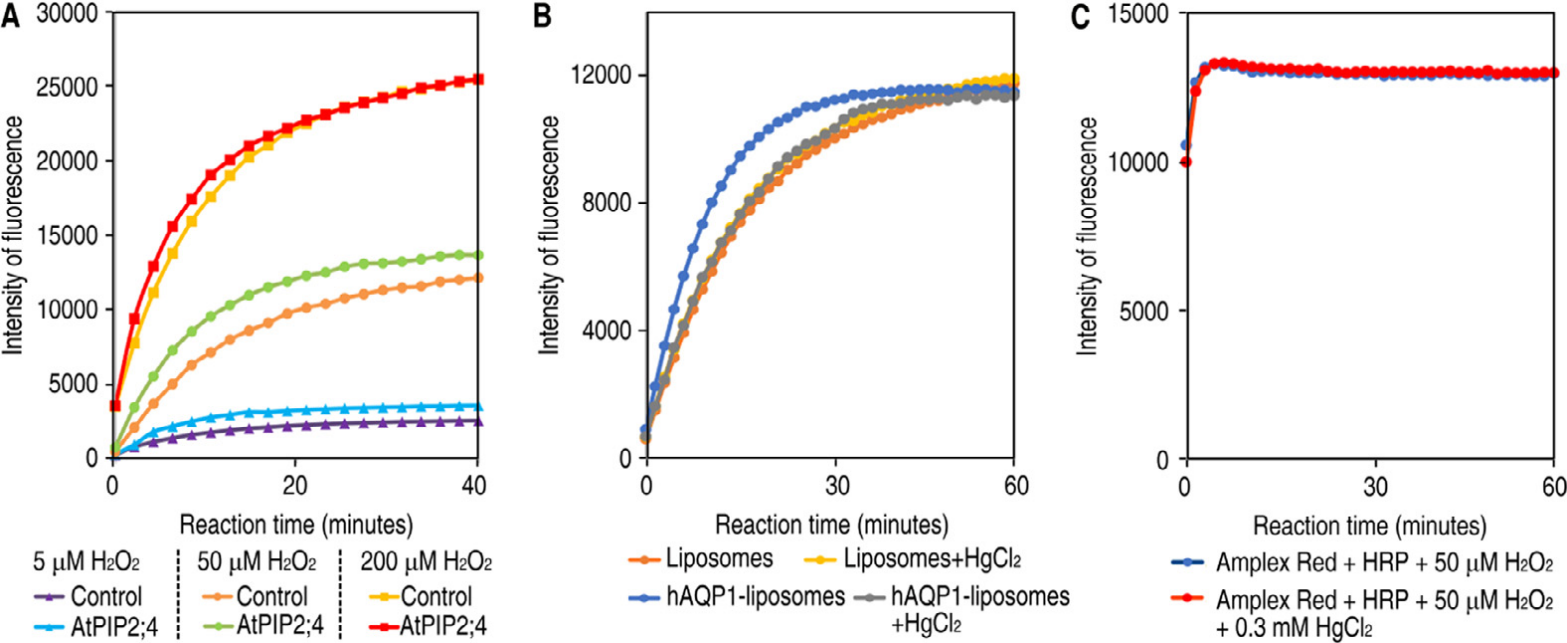
Confirming protein specific H_2_O_2_ transport in proteoliposomes. (A) The fluorescent intensity curves of empty liposomes and AtPIP2;4-liposomes treated with 5 µM, 50 µM and 200 µM H_2_O_2_, respectively. (B) The fluorescent intensity curves of liposomes and hAQP1 containing proteoliposomes, with or without mercury added. (C) Control experiment for the detection system of hydrogen peroxide mixed with or without mercury. *Figure adapted from Wang et al 2020.*

To establish the method, different concentrations of H_2_O_2_ was evaluated to define conditions giving a clear signal to background as well as a distinction between the protein mediated transport and simple diffusion over the lipid bilayer. Using a low concentration of H_2_O_2_, 5 µM, resulted in a low fluorescent signal and a small difference between the liposomes and proteoliposomes, respectively. A high H_2_O_2_ concentration (200 µM), on the other hand, resulted in a fast increase of the fluorescent intensity with the concomitant risk to cover the difference in transport between liposomes and proteoliposomes. Noteworthy, using 50 µM H_2_O_2_ gave an intermediate velocity development for the fluorescent signal and also an acceptable ratio of signal to background, this concentration was therefore selected for all measurement of aquaporin mediated H_2_O_2_ transport as well as for the negative control with empty liposomes (Fig. 3A).

### Confirming protein specific H_2_O_2_ transport

It is always possible that the actual reconstitution of a protein into the liposome alter the lipid bilayer and makes it leaky, giving the risk for misinterpretation of the transport in proteoliposomes. To exclude this possibility, and confirm that the observed H_2_O_2_ transport indeed was protein specific, an inhibitor for aquaporin was added to the proteoliposome preparation. Mercury is a well-established blocker for selected aquaporins, among those hAQP1, and this compound was therefore selected as inhibitor in the H_2_O_2_ transport assay. Noteworthy, by mercury addition, the H_2_O_2_ transport was fully abolished in the hAQP1 containing proteoliposomes and the curve overlaid the curve for the liposomes. To exclude that mercury had a negative effect on the lipid bilayer itself, a control experiment was performed by adding mercury to the empty liposomes, but no effect was observed confirming that the effect of mercury was protein specific (Fig. 3B). As an additional control experiment, a putative interference between mercury and the fluorescent reaction provided by AR, HRP and H_2_O_2_ was also investigated. Since no background reaction was observed, it was concluded that mercury is a compatible compound with the assay (Fig. 3C) and, over all, it was concluded that the observed increase of H_2_O_2_ transport in proteoliposomes was specifically due to the reconstituted aquaporin.

### Applicability of the assay for different aquaporin isoforms

**Fig. 4:**
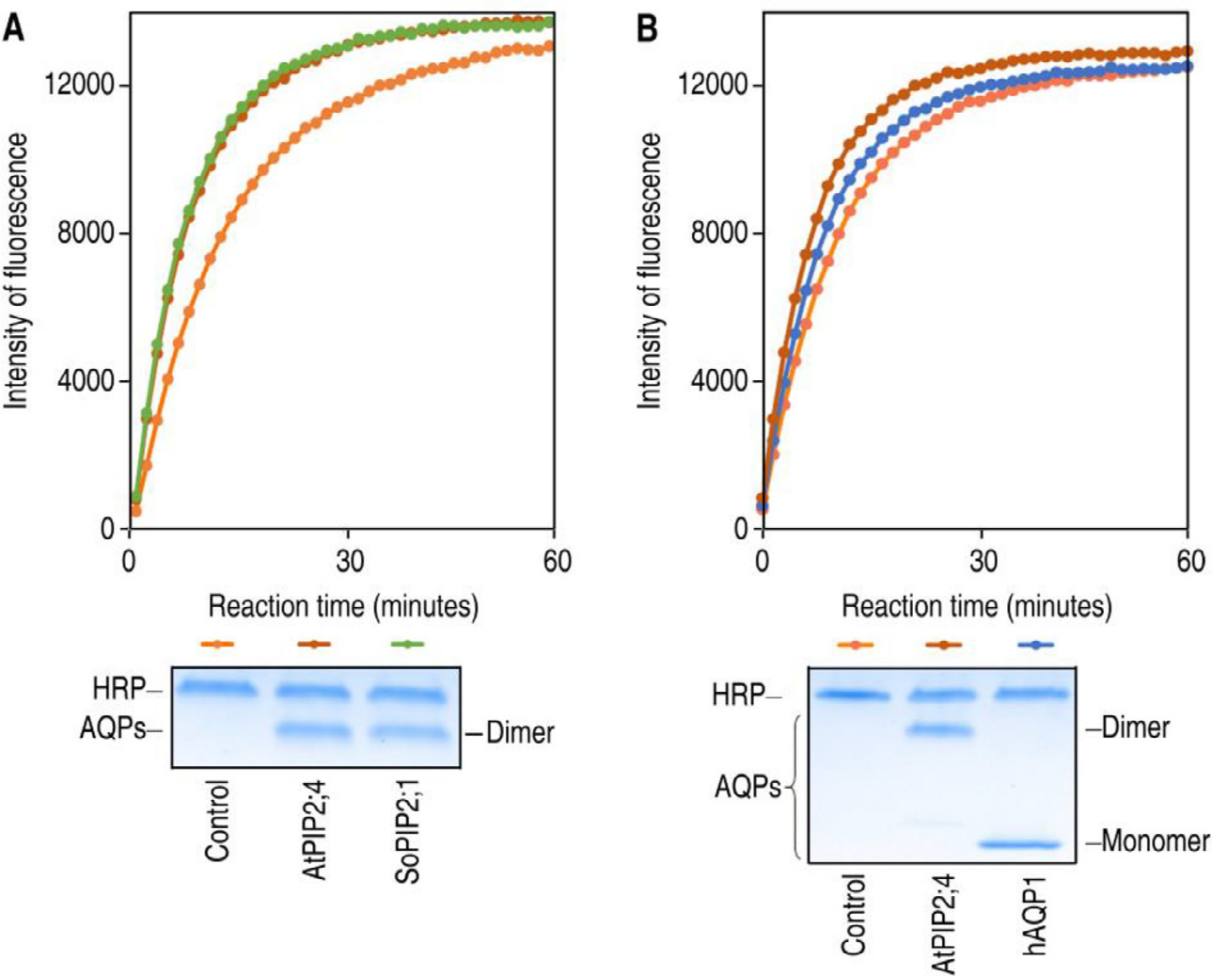
Applicability of the assay for different aquaporin isoforms. (A) Comparing hydrogen peroxide transport for two aquaporin isoforms from plant, AtPIP2;4 and SoPIP2;1, respectively. (B) Comparing hydrogen peroxide transport for two aquaporin isoforms from different organisms, AtPIP2;4 and hAQP1, respectively. Fluorescent intensity curves as well as SDS-PAGE analysis are shown for each experiment. *Figure adapted from Wang et al 2020.*

In a first comparison, two highly homologues aquaporins from plant, SoPIP2;1 and AtPIP2;4, were evaluated using the H_2_O_2_ assay. Both aquaporins showed efficient transport of H_2_O_2_ and the fluorescence was clearly higher than the one in empty liposomes. To confirm that the observed transport was due to comparable amounts of reconstituted protein, the proteoliposome preparations were analyzed by SDS-PAGE (Fig. 4A). To compare aquaporins from different organisms, AtPIP2;4 was compared to hAQP1 in the same experiment. Interestingly, a difference in H_2_O_2_ transport was observed for these two aquaporins. Again, the proteoliposomes were analysed by SDS-PAGE, excluding that the difference in transport efficiencies were due to different amounts of reconstituted protein (Fig. 4B). Noteworthy, the result on aquaporin homologues from plant and human, respectively, indicated that this assay has the potential to compare differences in the capacity of H_2_O_2_ transport between different targets.

### Comparison of the relative initial rate of H_2_O_2_ transport

The H_2_O_2_ assay is efficient in the sense that many samples can be compared simultaneously using the 96-well format. To quantitate the transport, the initial rate was evaluated for each target, having a minimum of three repeats. The Initial Rate (IR) was calculated by the equation

**Fig. 5:**
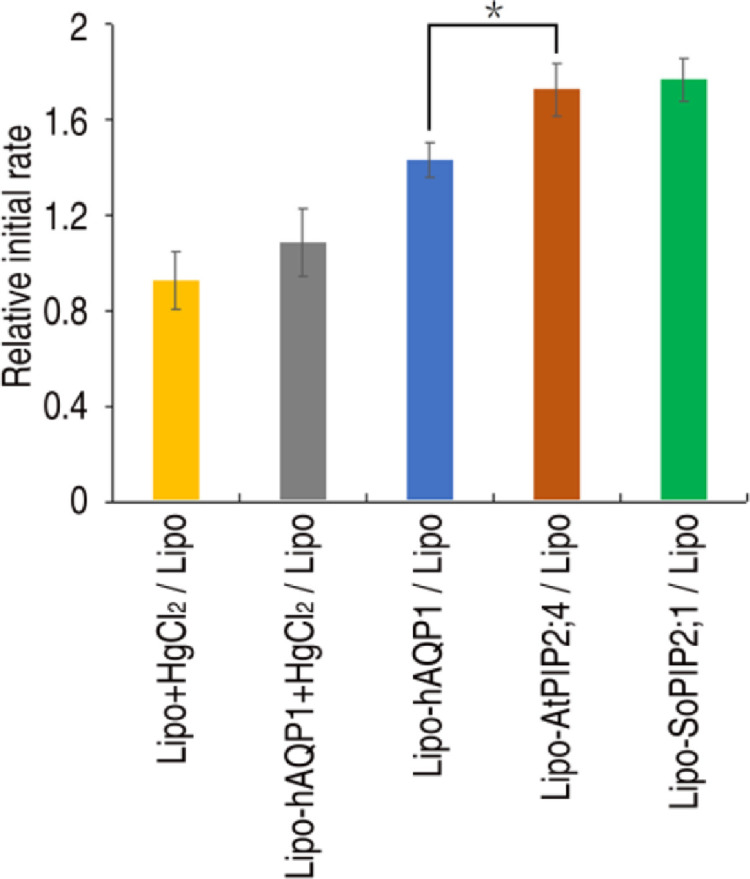
Comparison of the relative initial rate of H_2_O_2_ transport. Columns of relative initial rate (n = 3-6) for various aquaporin homologues are shown where ⁎ indicates significant difference. *Figure adapted from Wang et al 2020.*

IR=dF/dt

where F(t) is the function fitted to the curve, F(0) is the fluorescent intensity at 0 min and t is the time in minutes. Due to slight deviations in pipetting of the H_2_O_2_ solution, there are minor variations between the rates observed for samples measured in different plates, an aspect difficult to avoid. Since the relative signal is consistent between different plates, however, relative initial rates were calculated for each sample by calculating the ratio of the initial rate of proteoliposomes to that of empty liposomes within the same plate. Using this approach, there is no theoretical limit for the number of samples that could be compared, all together providing a robust way to compare differences in H_2_O_2_ permeability among aquaporins. Further, to evaluate the significance of different transport efficiencies, ANOVA-test is applied giving that a difference is significant for a *p* value < 0.05 (Fig. 5).

## Discussion

The method we established has been validated as a useful way to compare the capacity of various aquaporin isoforms in transporting H_2_O_2_ *in vitro*. Traditional methods for measuring H_2_O_2_ transport are normally based on living cell systems where expressing the aquaporins *in vivo* constitute an indispensable step. However, variations in production levels of individual protein targets as well as unavoidable interferences in the living cells make it non-trivial to accurately compare transport capacities between targets. Noteworthy, our method overcomes these disadvantages by reconstituting purified aquaporins in artificial liposomes.

The chemiluminescent probe used in the setup presented here is Amplex Red which is transformed to resorufin, catalyzed by HRP in the presence of H_2_O_2_. In fact, there are alternative fluorescent probes for detecting H_2_O_2_ which are established in cell-based assays. One of the most common probes to detect intracellular H_2_O_2_ is 2′,7′- Dichlorofluorescein diacetate (DCFH-DA) [9]. DCFH-DA permeates the cell membrane and is transformed to DCFH in the cell, catalyzed by esterase. However, in comparison to many other fluorescent probes, the transformation of DCFH to its fluorescent form requires the participation of additional compounds, like O_2_, which cannot be controlled [4], and hereby we regard DCFH-DA as not suitable for our assay since problems with specificity and reproducibility can be expected. Another common chemical probe for hydrogen peroxide measurements is based on aryl boronate. Boronate esters of polycyclic aromatic compounds interact with H_2_O_2_ and release a highly fluorescent product [10,11]. However, the boronate esters a membrane permeable which makes it difficult to keep them inside the liposomes. A possible solution to this could be to bind the boronate esters to a protein target or some other impermeable compound, a reaction that might be difficult and costly. An additional disadvantage with the boronate esters is that the reaction between H_2_O_2_ is irreversible and that could possibly lead to inaccurate quantification of transient fluxes of hydrogen peroxide [12].

Furthermore, there are protein based fluorescent probes for H_2_O_2_ based on enginereed proteins, including the commonly used HyPer. HyPer is based on the bacterial H_2_O_2_-sensing transcription factor OxyR and the circularly permuted yellow fluorescent protein (cpYFP) genetically inserted into the regulatory domain of OxyR [1]. The conformation of HyPer is altered by H_2_O_2_ which affects the disulfide bounds in the protein. A consequence is a shift in the fluorescent read out to an intensity at 500 nm. The fluorescent shift can also be detected *in vitro* by purified HyPer [1] and the protein can easily be encapsulated in liposomes, possibly providing an alternative system for hydrogen peroxide measurements. However, one concern to reflect upon is the necessity to keep the thiol group of HyPer reduced when encapsulated into liposomes, just to avoid the risk of disulfide bond formation in HyPer as a result from the interaction with H_2_O_2_. In the validation of this system, it would therefore be advisable to include an antioxidant, like the widely used β-mercaptoethanol, being aware of the paradox that the antioxidant itself could interfere with the reaction between HyPer and H_2_O_2_. Pointing this out, we do not exclude that HyPer could be a promising H_2_O_2_ sensor in the proteoliposome assay and it would be worth evaluating if this probe could improve the quantitative assay even further.
